# Maqui as a Chilean Functional Food: Antioxidant Bioactivity, Nutritional Value, and Health Applications

**DOI:** 10.3390/antiox15020204

**Published:** 2026-02-03

**Authors:** Caterina Tiscornia, Enrique Lorca, Carolina Estremadoyro, Valeria Aicardi, Fabián Vásquez

**Affiliations:** 1 Escuela de Nutrición y Dietética, Universidad Finis Terrae, Santiago 7501015, Chile; ctiscornia@uft.cl (C.T.); cestremadoyro@uft.cl (C.E.); 2 Escuela de Enfermería, Universidad Finis Terrae, Santiago 7501015, Chile; elorca@uft.cl; 3 Escuela de Kinesiología, Facultad de Arte y Educación Física, Universidad Metropolitana en Ciencias de la Educación, Santiago 7760170, Chile; 4 Unidad de Diálisis, Clínica Indisa, Santiago 7501014, Chile; valeria.aicardi@gmail.com

**Keywords:** *Aristotelia chilensis*, anthocyanins, delphinidins, oxidative stress, inflammation, antioxidants

## Abstract

Maqui (*Aristotelia chilensis*) is a berry native to southern Chile, recognized for its high content of phenolic compounds, particularly delphinidin-type anthocyanins, which confer strong antioxidant and anti-inflammatory properties and have generated growing interest as a functional food. Its scientific relevance has increased due to advances in understanding its biological mechanisms, including the Nrf2 signaling pathway, modulation of systemic inflammation, improvement in mitochondrial function, and potential applications in cardiometabolic, renal, and vascular health. **Objective:** The objective of this study is to analyze the available evidence on maqui in relation to its nutritional composition, bioactive profile, antioxidant and anti-inflammatory mechanisms, bioavailability, and emerging clinical applications in the prevention and/or treatment of chronic non-communicable diseases. **Main findings:** Maqui is rich in delphinidins, dietary fiber, and antioxidant micronutrients and modulates key oxidative stress and inflammatory pathways, including Nrf2-HO-1 and NF-κB. Preclinical and early clinical evidence supports its cardiometabolic and nephroprotective effects, with improvements in glycemic control, lipid metabolism, oxidative stress, and endothelial function. **Conclusions:** Maqui shows considerable potential as a Chilean functional food with antioxidant and anti-inflammatory effects relevant to human health. However, robust clinical trials and formulations with enhanced bioavailability are required to consolidate its therapeutic application.

## 1. Introduction

Maqui is a shrub species endemic to south-central Chile and an important part of the Mapuche biocultural heritage, where it has traditionally been used for food and medicinal purposes. In recent decades, its growing scientific and industrial value has positioned maqui as one of the Chilean wild fruits of the greatest nutritional and functional interest, both nationally and internationally, due to its exceptional density of bioactive compounds, particularly polyphenols [1,2].

From a phytochemical point of view, maqui stands out for its high content of anthocyanins, mainly derived from delphinidin (delphinidin-3-glucoside and delphinidin-3-sambubioside), which differentiates it from other widely studied berries such as blueberries and raspberries [3,4]. This composition gives maqui outstanding antioxidant capacity, with high stability in food matrices and standardized extracts, which has favored its development as a functional and nutraceutical ingredient [5].

At the molecular level, maqui anthocyanins actively participate in the regulation of cellular redox metabolism, exerting both direct and indirect antioxidant effects. The mechanisms described include the neutralization of reactive oxygen species (ROS), the activation of the Nrf2-ARE pathway, the inhibition of NF-κB, the modulation of endogenous antioxidant enzymes, and effects on mitochondrial function, chronic inflammation, and cellular senescence processes [6,7,8].

The role of dietary polyphenols in human health has been widely documented. Accumulated evidence shows that these compounds exert vasodilatory, anti-inflammatory, antilipemic, antiapoptotic, and endothelium-protective effects, attributable not only to their intrinsic antioxidant capacity but also to their interaction with cellular signaling pathways and key enzymes of oxidative metabolism [9,10]. They have been described as having the ability to reduce LDL oxidation, modulate the bioavailability of NO, regulate eNOS activity, and decrease platelet activation, which are central mechanisms in the prevention of cardiovascular disease [11].

Over the last decade, preclinical studies and initial clinical trials have explored the functional potential of maqui in various cardiometabolic conditions. In the renal field, a recent review has highlighted the possible nephroprotective role of its anthocyanins, showing reductions in oxidative stress, inflammation, and fibrosis in models of chronic kidney disease, along with improvements in metabolic and oxidative parameters in human studies [12]. Complementarily, a comprehensive review of the cardioprotective effects of maqui describes improvements in endothelial function, lipid profile, and ON bioavailability and a reduction in systemic inflammatory mediators, effects mainly attributed to its delphinidins [13]. Likewise, emerging evidence has been reported on antidiabetic effects, modulators of insulin resistance and systemic oxidative stress, consolidating maqui as a functional food of growing biomedical interest [14,15].

Considering these advances and considering recent progress in understanding the bioavailability of polyphenols, their active metabolites, and technological strategies aimed at improving their stability and biological efficacy, an integrated and updated synthesis of the available evidence is necessary.

The purpose of this review is to integrate the profile of bioactive compounds, the molecular mechanisms (antioxidant and anti-inflammatory), the available clinical evidence and limitations, formulations with greater bioavailability and therapeutic applications in cardiometabolic health.

## 2. Materials and Methods

This narrative review was structured following a systematic and transparent approach to ensure methodological rigor and reproducibility throughout the review process. A comprehensive literature search was conducted between August and December 2025 using the electronic databases PubMed, Scopus, ScienceDirect, Google Scholar, and MEDLINE, complemented by consultation of university library resources and relevant reference texts.

The search strategy employed Medical Subject Headings (MeSH) terms and Boolean operators combining keywords related to maqui and its biological effects, including:

“*Aristotelia chilensis*” OR “maqui berry” AND “anthocyanins” OR “delphinidins” AND “antioxidant activity” OR “oxidative stress” OR “inflammation” AND “Nrf2” OR “NF-κB” OR “mitochondrial function” OR “bioavailability” OR “polyphenols” OR “functional food” OR “nutraceuticals”.

To ensure comprehensive coverage, the reference lists and “cited by” sections of key articles were manually screened to identify additional relevant studies.

The inclusion criteria were as follows: (1) studies published up to December 2025; (2) original research articles, narrative or systematic reviews, and meta-analyses conducted in humans, animal models, or relevant in vitro systems; (3) publications addressing the nutritional composition, phytochemical profile, antioxidant and anti-inflammatory mechanisms, bioavailability, metabolism, or health-related applications of maqui or its bioactive compounds; and (4) articles published in English or Spanish. Exclusion criteria comprised publications without full-text access, duplicate records, studies not directly related to *Aristotelia chilensis*, and non-peer-reviewed or non-indexed sources.

The selection process involved an initial removal of duplicate records, followed by title and abstract screening to assess relevance. Full-text articles were subsequently evaluated to confirm compliance with inclusion criteria. Data extraction was performed in a standardized manner and included: study design, year of publication, experimental model or population characteristics, type and form of maqui or anthocyanin intervention, duration of exposure, evaluated outcomes (oxidative stress markers, inflammatory mediators, antioxidant enzymes, mitochondrial function, metabolic parameters), and principal findings. When available, information regarding dosage, bioavailability, safety, and reported adverse effects was also recorded.

The retrieved evidence was organized into thematic categories encompassing botanical and ethnobotanical background, nutritional and phytochemical composition, molecular antioxidant and anti-inflammatory mechanisms, bioavailability and metabolism, health applications, and technological and functional food development. Studies were further grouped according to experimental level (in vitro, preclinical, or clinical), and a critical narrative synthesis was performed based on methodological quality, biological plausibility, and translational relevance.

Discrepancies among authors regarding study inclusion or interpretation were resolved through consensus discussions to enhance consistency and validity. Technical terminology was carefully selected to facilitate multidisciplinary understanding while maintaining conceptual precision and minimizing redundancy, in accordance with the editorial standards of *Antioxidants*.

## 3. Botanical and Ethnobotanical Background of *Aristotelia chilensis*

### 3.1. Taxonomy and Distribution 

*Aristotelia chilensis*, commonly known as maqui, is a dioecious evergreen shrub or small tree belonging to the family Elaeocarpaceae. This family comprises more than 670 species distributed mainly across tropical and temperate regions of the Southern Hemisphere, with the genus *Aristotelia* represented by eight species occurring in South America and Oceania [2,3]. *A. chilensis* is endemic to the temperate forests of Chile and Argentina and is considered one of the most ecologically and culturally relevant native berry-producing species in southern South America [2,5].

### 3.2. Botanical and Ecological Characteristics

In Chile, *A. chilensis* exhibits a broad latitudinal distribution, ranging from the semi-arid Mediterranean zone of Coquimbo to the humid temperate ecosystems of the Aysén Region, occurring from sea level up to approximately 2500 m above sea level [4,6]. The species typically grows in humid environments such as ravines, riverbanks, forest edges, and disturbed areas, where it often forms dense stands known as *macales*. These formations play a critical ecological role in soil stabilization and early successional dynamics following disturbances such as fire or logging [5,7].

Botanically, maqui is characterized by a slender trunk, smooth gray bark, and opposite, serrated leaves with a coriaceous texture. The species is functionally dioecious, with male and female flowers borne on separate individuals, although incomplete dioecy has been reported, suggesting an ongoing evolutionary transition from hermaphroditism [8,9]. Flowering typically occurs between October and November, while fruiting takes place from December to February, depending on latitude and climatic conditions [6,7].

The fruit is a small, dark purple to black berry rich in anthocyanins, which not only confer its characteristic pigmentation but also facilitate zoochorous dispersal, primarily by birds. This dispersal mechanism contributes to the rapid colonization capacity of *A. chilensis* in disturbed habitats, reinforcing its role as a pioneer species in temperate forest ecosystems [6,10].

#### Climatic Requirements and Cold Tolerance

*Aristotelia chilensis* thrives in a range of temperate environments and shows remarkable ecological plasticity, growing from sea level up to 2500 m above sea level across central and southern Chile. Optimal climatic conditions for its growth include moderate temperatures between 10 °C and 20 °C, high humidity, and well-drained soils. The species performs best in areas with annual precipitation between 800 and 2000 mm, often in shaded or semi-shaded locations near watercourses or forest edges. The minimum temperature for vegetative activity is around 5 °C, below which growth significantly slows or halts. Although it is an evergreen species, *A. chilensis* has limited frost tolerance and can generally withstand short exposures to temperatures as low as –5 °C; however, temperatures below this threshold approach the freezing tolerance limit of vegetative tissues, beyond which irreversible cellular damage may occur, potentially affecting bud viability and subsequent fruit production. These thermal thresholds are particularly relevant for interpreting ecophysiological responses related to anthocyanin biosynthesis, especially delphinidin-based anthocyanins, and stress resilience [16,17].

### 3.3. Botanical Standardization and Fruit Maturity Considerations

*Aristotelia chilensis* is widely distributed across diverse ecological zones in Chile, resulting in significant variability in phenolic profiles influenced by geographic origin, environmental conditions, and fruit maturity. Although no formally recognized subspecies have been described, substantial intraspecific variation has been documented, particularly in anthocyanin content and antioxidant capacity. Consequently, this review considered only studies that clearly identified the botanical source of maqui samples and, when possible, reported fruit maturity stage and phytochemical characterization. Emphasis was placed on investigations using standardized or chemically defined extracts to minimize variability and improve biological comparability. Fruit maturity was considered a critical determinant of biological activity, as fully ripe berries consistently exhibit higher concentrations of delphinidin-based anthocyanins and greater antioxidant potential than unripe stages [17,18,19].

### 3.4. Phytochemical Composition of Maqui Fruit

The distinctive biological properties attributed to maqui are underpinned by its unique phytochemical profile. In order to address the specificity of the fruit composition, Table 1 summarizes the main polyphenolic compounds identified in ripe *Aristotelia chilensis* fruit, including total phenolics, total anthocyanins, and individual anthocyanin species, together with other relevant polyphenolic subclasses. The data presented are derived from well-characterized fruit matrices and highlight the predominance of delphinidin-based anthocyanins, which define the characteristic antioxidant signature of maqui.

Values are expressed as ranges to reflect natural variability associated with geographic origin, fruit maturity, and processing conditions. Data correspond to ripe maqui fruit and are reported on a fresh weight (FW) or dry weight (DW) basis, as specified. Total phenolic content was determined using the Folin–Ciocalteu method and expressed as gallic acid equivalents (GAE), while total anthocyanins were quantified as cyanidin-3-glucoside (C3G) equivalents. Individual anthocyanins and other polyphenols were identified and quantified by chromatographic techniques. The predominance of delphinidin-based anthocyanins represents the distinctive phytochemical feature of *Aristotelia chilensis* fruit [17,19,20].

It is noteworthy that, compared with other anthocyanin-rich berries commonly studied for their biological activity, maqui exhibits a distinctive phytochemical and functional profile. Although berries such as blueberry, açaí, and Calafate are also recognized for their antioxidant and anti-inflammatory properties, their anthocyanin composition differs markedly from that of maqui. In particular, maqui is characterized by an unusually high proportion of delphinidin-based anthocyanins, whereas cyanidin derivatives predominate in cranberry and blueberry, and malvidin and peonidin derivatives are more abundant in matrices derived from blueberry and grape [20,21,22].

These comparative data indicate that, while several berries share overlapping biological properties, the predominance of delphinidin-based anthocyanins confers a unique functional identity to maqui. From a functional perspective, these compositional differences translate into distinct biological effects [20,21,22].

In this context, the Table 2 summarizes and compares the main biological properties of maqui with those of other berries exhibiting similar functional attributes.

### 3.5. Ethnobotanical Significance and Traditional Uses

From an ethnobotanical perspective, *A. chilensis* holds profound cultural significance among Indigenous peoples of southern Chile, particularly the Mapuche. Known locally as *maqui*, *klon* or *queldrón*, the plant is regarded as a sacred species associated with benevolence, healing, and spiritual protection. Traditional Mapuche cosmology attributes a *ngen* (spiritual guardian) to the plant, and ritual respect is required prior to harvesting its fruits or leaves [11].

Historically, maqui fruits were consumed fresh or processed into fermented beverages such as *tecu*, while leaves and bark were widely used in traditional medicine. Ethnomedicinal applications include treatment of gastrointestinal disorders, inflammatory conditions, wounds, sore throat, and fever, employing infusions, poultices, or topical preparations derived from leaves and fruits [3,12]. These ancestral uses provided the empirical foundation for later pharmacological and phytochemical research, which has validated several of these traditional claims [13,14].

In recent decades, growing scientific and commercial interest in maqui has repositioned *A. chilensis* as a high-value nutraceutical species. Nevertheless, its traditional ethnobotanical context remains central to understanding sustainable harvesting practices, conservation priorities, and ethical considerations regarding biocultural heritage. The integration of botanical knowledge with Indigenous ecological understanding is therefore essential for the responsible development of maqui-based products and for safeguarding the ecological and cultural integrity of native forest systems [6,7].

## 4. Nutritional Composition and Bioactive Profile

Maqui is a native berry from the central-southern region of Chile and is recognized for its exceptional nutritional and phytochemical profile. The fruit contains a diverse array of bioactive compounds that position it as a high-value functional food.

### 4.1. Macronutrients and Micronutrients

The maqui berry is low in calories and rich in dietary fiber, contributing to gastrointestinal health. It provides modest amounts of carbohydrates and contains negligible levels of fats and proteins. Maqui also delivers essential micronutrients, including potassium, calcium, magnesium, and vitamin C, which play roles in electrolyte balance and antioxidant defense [15].

### 4.2. Anthocyanins and Phenolic Compounds

The most studied constituents of maqui are its anthocyanins, primarily delphinidin-3-O-glucoside, delphinidin-3,5-O-diglucoside, and cyanidin derivatives, which contribute to its deep purple coloration and strong antioxidant potential [27,28]. These anthocyanins exhibit high radical-scavenging activity and are more abundant in maqui than in other commonly consumed berries such as blueberries or blackberries [29].

In addition to anthocyanins, maqui contains flavonoids, phenolic acids (e.g., gallic acid, ellagic acid), and stilbenes, which collectively contribute to its anti-inflammatory, vasodilatory, and antidiabetic effects [30,31].

### 4.3. Antioxidant Capacity and Stability

**Figure 1:**
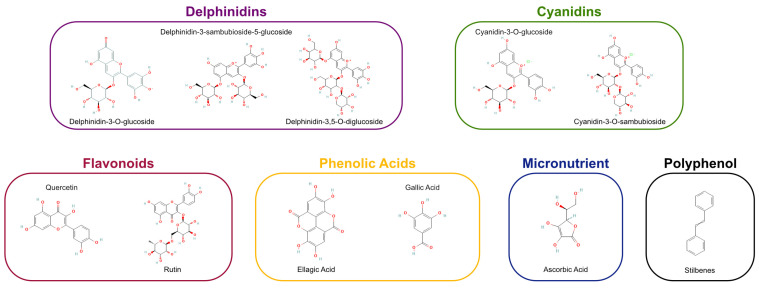
Profile of antioxidant classes and specific bioactive agents in *Aristotelia chilensis*. Schematic representation categorizing the essential antioxidants found in Maqui berries. The classification encompasses delphinidins, cyanidins, flavonoids, phenolic acids, micronutrients, and stilbenes, identifying the key molecules responsible for the fruit’s oxidative stress modulation properties.

Maqui extracts have demonstrated superior antioxidant activity in multiple assays, including DPPH (2,2-Diphenyl-1-picrylhydrazyl), ORAC (Oxygen Radical Absorbance Capacity), and ABTS (2,2′-Azino-bis (3-ethylbenzothiazoline-6-sulfonic acid), surpassing that of other berries [1]. Furthermore, anthocyanins from maqui show notable chemical stability, which enhances their utility in functional foods, nutraceuticals, and beverages [32] (Figure 1).

The method used to prepare maqui berry extracts significantly influences the chemical profile and concentration of bioactive compounds detected, particularly anthocyanins and total phenolics. Most antioxidant and phytochemical analyses in the literature rely on hydroalcoholic extraction, commonly employing mixtures such as ethanol: water (70:30 or 80:20 *v*/*v*) or methanol: water acidified with 0.1% HCl to optimize anthocyanin solubilization. These solvent systems have proven effective for recovering delphinidin-based anthocyanins, the dominant phenolics in maqui [17,33].

In recent studies, UAE and MAE have been applied to improve extraction efficiency, selectivity, and compound stability. These advanced methods allow for better preservation of thermolabile compounds and may yield extracts with different biological activity profiles [34,35].

Moreover, post-extraction processes such as lyophilization (freeze-drying), filtration, and storage temperature (e.g., −20 °C or vacuum-sealed conditions) are critical for maintaining anthocyanin integrity and antioxidant potential during storage and analysis. Therefore, interpretation of antioxidant capacity results must consider the specific extraction and handling protocols employed, as comparisons across studies using different methods may not be directly equivalent. Importantly, delphinidin-based anthocyanins display marked pH-dependent stability, being the most stable under acidic conditions (pH ≤ 3) while undergoing structural degradation and color loss at neutral or alkaline pH. This pH sensitivity directly affects both the measured antioxidant capacity and the functional performance of maqui extracts in food matrices and biological systems [34,35,36].

Therefore, interpretation of antioxidant activity data must carefully consider the extraction method, post-processing conditions, and pH environment, as studies employing different protocols are not strictly comparable in quantitative terms. Recognizing these methodological variables is essential for accurately contextualizing the antioxidant potential and technological applicability of maqui-derived products.

### 4.4. Emerging Bioactivities

Recent studies suggest that maqui polyphenols modulate key signaling pathways such as Nrf2, NF-κB, and AMPK, impacting mitochondrial biogenesis, inflammation resolution, and metabolic regulation [37,38]. These molecular properties support the potential use of maqui in interventions for metabolic syndrome, cardiovascular diseases, and chronic kidney disease.

## 5. Antioxidant and Anti-Inflammatory Mechanisms of Action

Oxidative stress and chronic inflammation represent fundamental pathophysiological processes underlying numerous age-related and metabolic diseases, including CVD, diabetes, neurodegenerative disorders, and cancer [39,40,41]. The Nrf2 and nuclear NF-κB signaling pathways have been identified as master regulators of cellular responses to oxidative and inflammatory insults, respectively [42,43,44]. Therapeutic strategies that target these pathways have attracted considerable interest in the development of preventive and treatment approaches for chronic diseases.

Recent scientific investigations have revealed that maqui berry possesses remarkably elevated antioxidant capacity, primarily ascribed to its abundant presence of anthocyanins, particularly delphinidin-3-sambubioside-5-glucoside and delphinidin-3,5-diglucoside, along with other polyphenolic compounds including quercetin, rutin, and various phenolic acids [33,34,35,36,37,38,39,40,41,42,43].

The mounting body of evidence indicates that maqui berry extracts elicit their salutary effects through the modulation of pivotal molecular pathways implicated in cellular defense and inflammation [44,45]. However, a comprehensive synthesis of the specific molecular mechanisms, particularly regarding Nrf2 pathway activation (including downstream targets HO-1, NQO1, SOD, GPx), ROS reduction, NF-κB pathway inhibition (including TNF-α, IL-6, COX-2), mitochondrial function, and cellular senescence, remains lacking in the literature.

### 5.1. Bioactive Constituents of Aristotelia chilensis

The therapeutic potential of *Aristotelia chilensis* is primarily determined by the qualitative and quantitative predominance of specific polyphenolic subclasses, rather than by total polyphenol content alone. Among these, anthocyanins (particularly delphinidin-derived glycosides) constitute the main bioactive substrates responsible for the biological effects described in subsequent sections.

Phytochemical analyses consistently demonstrate that maqui berry contains exceptionally high concentrations of anthocyanins compared with other edible berries, with delphinidin derivatives accounting for approximately 70–80% of total anthocyanin content [17,33,43,44].

The major compounds include delphinidin-3-sambubioside-5-glucoside, delphinidin-3,5-diglucoside, cyanidin-3-sambubioside-5-glucoside, and cyanidin-3,5-diglucoside. This distinctive anthocyanin profile is of particular relevance, as delphinidin glycosides exhibit higher antioxidant capacity and redox-modulating activity than cyanidin-based anthocyanins, largely due to the increased number of hydroxyl groups on the B-ring structure.

From a mechanistic standpoint, anthocyanin-rich profiles characterized by a high proportion of delphinidin-based glycosides are consistently associated with, and are likely major contributors to, the antioxidant, anti-inflammatory, mitochondrial, and signaling effects attributed to maqui. These compounds have been shown to contribute to reactive oxygen species scavenging, support activation of endogenous antioxidant pathways (e.g., Nrf2-dependent transcription), and are implicated in the modulation of inflammatory signaling cascades (NF-κB, MAPK), mitochondrial function, and cellular senescence pathways, as detailed in Section 5.2, Section 5.3, Section 5.4, Section 5.5 and Section 5.6.

In addition to anthocyanins, maqui berry contains relevant amounts of other polyphenolic subclasses, including flavonols (quercetin, rutin, myricetin), phenolic acids (gallic and protocatechuic acids), and proanthocyanidins [17,46,47]. These compounds contribute complementary and synergistic biological effects, particularly through modulation of inflammatory mediators, enzyme inhibition, and reinforcement of antioxidant defenses. However, their biological activity appears to be more limited in scope with respect to redox-sensitive signaling and inflammatory pathway modulation when compared with delphinidin-rich anthocyanin profiles and is best interpreted as supportive rather than primary in the context of signaling transduction, mitochondrial regulation, and cytokine modulation.

Accordingly, while maqui berry exhibits a complex polyphenolic composition, the biological mechanisms described in Section Downstream Antioxidant Enzymes, Section 5.3, Section 5.4, Section 5.4.1, Section 5.4.2, Section 5.5 and Section 5.6 are largely associated with anthocyanin-rich profiles characterized by a high proportion of delphinidin-based glycosides, with other polyphenols acting as complementary modulators that enhance overall bioactivity.

Commercial maqui extracts, such as Delphinol^®^, are standardized to ensure high concentrations of delphinidin glycosides (≥25%) and total anthocyanins (≥35%), thereby providing reproducible biological effects across experimental and clinical studies [44,45]. The systemic actions of these compounds are further influenced by their bioavailability and metabolism, including intestinal absorption, phase II conjugation, and gut microbiota-mediated biotransformation, processes that ultimately determine their antioxidant and anti-inflammatory efficacy in vivo [43,46].

### 5.2. Nrf2 Pathway Activation and Antioxidant Defense

The Nrf2 pathway is a critical cellular defense mechanism against oxidative stress. Under basal conditions, Nrf2 is sequestered in the cytoplasm by Kelch-like ECH-associated protein 1 (Keap1), which promotes its ubiquitination and proteasomal degradation. Upon exposure to oxidative stress or electrophilic compounds, Nrf2 dissociates from Keap1, translocated to the nucleus, and binds to ARE in the promoter regions of cytoprotective genes [43].

A considerable body of research has demonstrated that maqui berry extracts, particularly those enriched in delphinidin-based anthocyanins, activate the Nrf2 signaling pathway in a variety of experimental models. In a 3D bone co-culture system exposed to CSE, MBE at 1.5 µg/mL significantly activated the Nrf2 signaling pathway, counteracting CSE-induced oxidative damage [40]. In a similar manner, in Caco-2 intestinal epithelial cells, maqui extracts activated Nrf2, thereby contributing to their anti-inflammatory and antioxidant effects [39].

In the context of inflammatory bowel disease, it has been demonstrated that polyphenolic maqui extract, with a high anthocyanin and delphinidin glycoside content, activates the Nrf2 pathway in a TNBS-induced Crohn’s disease mouse model, resulting in the upregulation of downstream antioxidant enzymes [43]. This Nrf2 activation was accompanied by increased nuclear translocation of Nrf2 protein and enhanced ARE-driven gene transcription [43].

#### Downstream Antioxidant Enzymes

The activation of Nrf2 by maqui berry extracts leads to coordinated upregulation of multiple antioxidant enzymes, creating a comprehensive cellular defense system:

- *Heme Oxygenase-1 (HO-1):* HO-1 is a critical Nrf2-regulated enzyme that catalyzes the degradation of heme to biliverdin, carbon monoxide, and free iron, providing potent cytoprotective effects. In a mouse model of Crohn’s disease, maqui extract significantly increased HO-1 expression in colonic tissue, contributing to reduced oxidative damage and inflammation [41,43]. The upregulation of HO-1 was also observed in bone cells exposed to cigarette smoke, where maqui extract prevented oxidative injury [33,40].
- *NAD(P)H Quinone Oxidoreductase 1 (NQO1):* NQO1 is a flavoenzyme that catalyzes two-electron reduction of quinones, preventing the generation of reactive semiquinone radicals. While direct measurement of NQO1 in maqui berry studies is limited in the available literature, the activation of Nrf2 pathway strongly suggests coordinated upregulation of NQO1 alongside other ARE-regulated genes [39,43].
- *Superoxide Dismutase (SOD):* SOD enzymes catalyze the dismutation of superoxide radicals to hydrogen peroxide and oxygen, representing the first line of defense against ROS. In a metabolic syndrome rat model, 14-day administration of maqui berry significantly improved serum SOD activity in both male and female rats subjected to a high-fat, high-fructose diet [47]. Similarly, in cigarette smoke-exposed osteoblasts, maqui extract enhanced SOD-1 expression, protecting against oxidative damage [33,46].
- *Glutathione Peroxidase (GPx):* The glutathione system, including GPx, glutathione reductase, and glutathione S-transferases, plays a central role in cellular antioxidant defense. Maqui berry extracts have been shown to enhance glutathione levels and GPx activity across multiple experimental models [43,46]. In the TNBS-induced colitis model, maqui extract restored glutathione content in colonic tissue, which was depleted by inflammatory insult [43].
- *Catalase:* Catalase catalyzes the decomposition of hydrogen peroxide to water and oxygen, working in concert with SOD to eliminate ROS. Enhanced catalase activity has been reported in various maqui berry intervention studies, contributing to overall antioxidant capacity [43,46].

### 5.3. ROS Reduction Mechanisms

The ultimate functional outcome of Nrf2 pathway activation is the reduction in intracellular ROS and mitigation of oxidative damage to cellular macromolecules. The potential of maqui berry extracts, particularly those enriched in delphinidin-based anthocyanins, to scavenge ROS has been demonstrated in a variety of experimental systems.

In bone cells exposed to cigarette smoke, delphinidin-rich maqui berry extract significantly reduced CSE-induced ROS generation, as measured by DCF fluorescence assays [40,41]. The ROS-reducing effects were found to be dose-dependent, with 1.5 µg/mL MBE providing optimal protection without evidence of toxicity [40].

In models of inflammatory bowel disease, anthocyanin-rich maqui extracts have been shown to significantly reduce oxidative stress markers. These include (MDA), a product of lipid peroxidation, and protein carbonyls, which are indicators of oxidative protein damage [43,47]. In the metabolic syndrome rat model, administration of maqui berry resulted in a reduction in serum MDA concentrations and carbonyl formation in both male and female animals [47].

In human smokers, a clinical study demonstrated that maqui berry extract intake normalized H_2_O_2_ concentrations in exhaled breath condensate, indicating reduced pulmonary oxidative stress [12]. This finding suggests that the ROS-reducing effects observed in preclinical models can be translated to human applications.

The photoprotective effects of maqui berry also involve a reduction in ROS. In keratinocytes exposed to UVB radiation, maqui extract significantly decreased UVB-induced ROS generation, thereby preventing oxidative damage to skin cells [13]. In a similar manner, in murine photoreceptor-derived cells exposed to blue light, delphinidin-rich maqui extract was found to ameliorate blue light-induced ROS production and subsequent organelle damage [48], further supporting the central role of delphinidin-based anthocyanins in ROS mitigation across different tissues.

### 5.4. NF-κB Modulation and Anti-Inflammatory Effects

The NF-κB pathway is a master regulator of inflammatory responses, controlling the transcription of numerous pro-inflammatory genes. Under resting conditions, NF-κB dimers are sequestered in the cytoplasm by inhibitory IκB proteins. Pro-inflammatory stimuli activate the IκB kinase (IKK) complex, leading to IκB phosphorylation, ubiquitination, and degradation, allowing NF-κB to translocate to the nucleus and activate target gene transcription [43].

The potential of delphinidin-rich maqui berry extracts to inhibit NF-κB signaling has been demonstrated in a variety of experimental models. In the 3D bone co-culture system exposed to cigarette smoke, maqui berry extract (1.5 µg/mL) significantly inhibited CSE-induced NF-κB activation, thereby preventing the upregulation of inflammatory mediators [40]. This inhibition manifested at various levels, including diminished IκB degradation and diminished nuclear translocation of NF-κB subunits [40,43]. In Caco-2 intestinal epithelial cells, maqui extracts have been shown to inhibit NF-κB signaling, thereby contributing to their anti-inflammatory effects [39]. In a similar manner, in the TNBS-induced Crohn’s disease model, anthocyanin-rich maqui extract, characterized by a high delphinidin glycoside content, suppressed NF-κB activation in colonic tissue, correlating with reduced expression of NF-κB-dependent inflammatory genes [43].

The mechanisms underlying NF-κB modulation by delphinidin-rich maqui berry extracts are likely multifactorial. These include direct antioxidant effects that limit ROS-mediated NF-κB activation, inhibition of upstream kinases such as IKK, and modulation of post-translational modifications of NF-κB subunits [43,46]. Collectively, these findings indicate that maqui berry extracts enriched in delphinidin-based anthocyanins are consistently associated with attenuation of NF-κB signaling under inflammatory conditions. However, the available evidence supports a profile-driven and context-dependent mechanism, in which the overall anthocyanin composition and redox-modulating capacity of the extract contribute to NF-κB regulation, rather than allowing for definitive attribution of these effects to delphinidin alone.

#### 5.4.1. Pro-Inflammatory Cytokine Modulation

Inhibition of NF-κB signaling by delphinidin-rich maqui berry extracts results in reduced expression and secretion of multiple pro-inflammatory cytokines and mediators.

- Tumor Necrosis Factor-α: TNF-α is a key pro-inflammatory cytokine that plays a central role in inflammatory diseases. In the TNBS-induced colitis model, anthocyanin-rich maqui extract, characterized by a high delphinidin glycoside content, significantly reduced colonic TNF-α levels, contributing to amelioration of intestinal inflammation [43]. In adipocyte-macrophage co-culture systems, maqui extract suppressed TNF-α secretion, reducing inflammatory crosstalk between these cell types [49].
- Interleukin-6: IL-6 is a pleiotropic cytokine involved in acute and chronic inflammation. Clinical evidence from healthy smokers demonstrated that intake of delphinidin-rich maqui berry extract normalized IL-6 concentrations in exhaled breath condensate, indicating reduced pulmonary inflammation [12]. In preclinical models, maqui extracts consistently reduced IL-6 expression in inflamed tissues [43,48].
- Interleukin-1β: IL-1β is a potent pro-inflammatory cytokine and a key component of the inflammasome pathway. In the Crohn’s disease model, anthocyanin-rich maqui extract reduced IL-1β levels in colonic tissue, contributing to resolution of inflammation [41,43].
- Cyclooxygenase-2: COX-2 is an inducible enzyme responsible for prostaglandin synthesis during inflammation. Multiple studies have demonstrated that delphinidin-rich maqui extracts suppress COX-2 expression through NF-κB inhibition [41,43,50]. In the TNBS-induced colitis model, maqui extract significantly reduced colonic COX-2 expression, correlating with decreased PGE_2_ production [43].
- Inducible Nitric Oxide Synthase: iNOS generates large quantities of nitric oxide during inflammation, contributing to oxidative and nitrosative stress. Maqui extracts have been shown to suppress iNOS expression under inflammatory conditions [41,43,50], resulting in reduced NO production and attenuation of tissue damage in colitis models [43].

Taken together, these findings indicate that anthocyanin-rich maqui berry extracts are consistently associated with broad suppression of pro-inflammatory mediators downstream of NF-κB signaling, reinforcing their role as modulators of inflammatory cytokine networks rather than as single-target inhibitors.

#### 5.4.2. Inflammasome Regulation

Beyond classical NF-κB signaling, increasing evidence suggests that anthocyanin-rich maqui berry extracts modulate inflammasome activation, particularly the NLRP3 inflammasome, a critical mediator of inflammatory and immune responses.

In a murine model of Crohn’s disease, an anthocyanin-rich maqui extract characterized by high delphinidin glycoside content inhibited NLRP3 inflammasome activation, as demonstrated by reduced expression of NLRP3, ASC (apoptosis-associated speck-like protein containing a CARD), and caspase-1, together with decreased maturation of IL-1β [41]. This suppression of inflammasome activation was associated with reduced intestinal inflammation and improved disease outcomes [41].

Mechanistically, inhibition of inflammasome activation by delphinidin-rich maqui extracts appears to involve complementary processes. These include attenuation of intracellular and mitochondrial ROS generation, preservation of mitochondrial integrity, and interference with inflammasome assembly and activation [41,43]. Overall, these observations support a role for anthocyanin-rich maqui berry extracts in the regulation of inflammasome-driven inflammation while emphasizing that inflammasome modulation likely arises from integrated redox and mitochondrial mechanisms rather than from a delphinidin-specific effect alone.

### 5.5. Mitochondrial Function and Protection

Mitochondria are pivotal to cellular energy metabolism and serve as significant sources of ROS under pathological conditions. Mitochondrial dysfunction has been implicated in numerous diseases, including metabolic disorders, neurodegenerative diseases, and aging [45,48].

Emerging evidence suggests that delphinidin-rich maqui berry extracts protect mitochondrial function damage under conditions of oxidative stress. In murine photoreceptor-derived cells exposed to blue light, delphinidin-rich maqui extract has been shown to ameliorate subcellular organelle damage, including mitochondrial dysfunction [48]. The extract was found to be effective in preventing blue light-induced mitochondrial membrane potential loss and maintaining mitochondrial integrity [48].

In the context of bone health, maqui berry extract has been shown to protect osteoblasts from mitochondrial dysfunction induced by cigarette smoke [33,40]. The extract was found to preserve mitochondrial membrane potential and to prevent CSE-induced mitochondrial ROS production, thereby contributing to the maintenance of cellular bioenergetics [40].

In a study of the impact of delphinidin-enriched maqui extract on bone metabolism, the extract was found to enhance mitochondrial function in both osteoblasts and osteoclasts. This, in turn, led to increased bone formation and reduced bone resorption in osteopenic mouse models [45]. The mitochondrial protective effects were associated with improved cellular ATP production and reduced oxidative damage to mitochondrial components [45].

The mechanisms underlying mitochondrial protection by delphinidin-based anthocyanins from maqui berry involve: (1) reduction in mitochondrial ROS through enhanced antioxidant enzyme expression, (2) preservation of mitochondrial membrane integrity, (3) maintenance of mitochondrial membrane potential, and (4) prevention of mitochondrial permeability transition pore opening [45,46].

Nevertheless, current evidence primarily supports an association between delphinidin-rich anthocyanin profiles and improved mitochondrial resilience, while detailed assessments of mitochondrial dynamics, biogenesis markers, and respiratory chain function remain limited, highlighting important directions for future research.

### 5.6. Anti-Cellular Senescence

Cellular senescence, characterized by irreversible cell cycle arrest, altered metabolism, and a senescence-associated secretory phenotype (SASP), contributes to the process of aging and the development of age-related diseases [45,51,52]. Despite the paucity of direct investigation of cellular senescence markers in studies using delphinidin-rich maqui berry extracts, a plethora of evidence suggests the potential for anti-senescence effects.

A delphinidin-enriched maqui extract has been demonstrated to offer protective effects against bone loss in osteopenic mouse models, a condition associated with age-related cellular senescence in bone tissue [45]. The extract demonstrated the capacity to enhance bone metabolism and to act as a safeguard against age-related bone deterioration, thereby indicating the possibility of regulating senescence pathways [45].

In the context of cutaneous aging, anthocyanin-rich maqui berry extracts have demonstrated potential for photoprotection and anti-aging effects [13,51]. UVB radiation has been identified as a significant inducer of cellular senescence in skin cells. Furthermore, it has been demonstrated that maqui extract protects keratinocytes from UVB-induced damage, thereby preventing premature cellular aging [13]. The photoprotective effects are characterized by a reduction in ROS, the prevention of DNA damage, and the maintenance of cellular proliferative capacity [13].

The potential anti-senescence mechanisms associated with delphinidin-based anthocyanins from maqui berry are likely to involve multiple, interrelated processes. These include: (i) reduction in oxidative stress, a key driver of cellular senescence; (ii) modulation of senescence-associated signaling pathways (p53, p16, p21); (iii) suppression of SASP factors through NF-κB inhibition; and (iv) maintenance of mitochondrial function, which is critical for delaying senescence [45,51,52].

Importantly, these findings should be interpreted as indicative of a potential anti-senescence capacity associated with the overall anthocyanin-rich profile of maqui, rather than as conclusive evidence for a delphinidin-specific mechanism. Direct quantification of senescence markers such as SA-β-gal, p16^INK4a, p21^CIP1, and SASP components remains an essential area for future investigation.

For greater clarity, Figure 1 shows the Profile of antioxidant classes and specific bioactive agents in *Aristotelia chilensis*.

## 6. Bioavailability, Bioaccessibility and Metabolism of Maqui Anthocyanins

Despite the growing interest in maqui berry as a functional food ingredient and dietary supplement, fundamental questions regarding the bioavailability, absorption, and metabolism of its anthocyanins remain incompletely answered. The apparent paradox between low systemic bioavailability (typically less than 1% of the ingested dose) and documented biological effects has driven research into absorption mechanisms, metabolic transformations, and strategies to enhance bioavailability [53]. It is imperative to comprehend these processes to optimize the health benefits of maqui anthocyanins and to formulate evidence-based recommendations for their consumption.

### 6.1. Bioavailability and Pharmacokinetics

#### 6.1.1. Human Clinical Studies

Human bioavailability studies of maqui anthocyanins have yielded pivotal insights into their absorption and pharmacokinetic profiles. Schön et al. [54] conducted a bioavailability study in 12 healthy subjects following single-dose supplementation with a standardized maqui berry extract (Delphinol^®^). The study tracked plasma concentrations of delphinidin-3-O-glucoside and cyanidin-3-O-sambubioside over a period of eight hours using a liquid chromatography-tandem mass spectrometry (LC-MS/MS) analysis. The results demonstrated rapid absorption, with maximum concentrations of DG occurring at 1.0 ± 0.3 h post-ingestion and corticosterone (CS) peaking at 2.0 ± 1.1 h. Plasma concentrations returned to within 8 h of baseline, indicating rapid clearance [54].

The pharmacokinetic parameters observed for maqui anthocyanins align with broader patterns documented for berry anthocyanins. In a comparative study of wild blueberry polyphenols, Zhong et al. [55] reported that parent anthocyanin peaked approximately 2 h post-ingestion, with bioavailability of wild blueberry anthocyanins at 1.1% and chlorogenic acid at 0.2%. Phase II metabolites, including glucuronide conjugates of delphinidin, cyanidin, peonidin, and petunidin, exhibited delayed peaks at 2.6, 7, 6.3, and 8.8 h, respectively, demonstrating biphasic absorption patterns [55].

Agulló et al. [56] investigated the presence of anthocyanin metabolites in human urine following acute ingestion of maqui-citrus functional beverages in a double blind, randomized, crossover study involving 20 overweight individuals. Urinary samples were collected at 0, 0–3.5 h, 3.5–12 h, and 12–24 h post-ingestion and analyzed by UHPLC-ESI-MS/MS. It is noteworthy that no parental anthocyanins were detected in the urine; instead, extensive degradation products and metabolites were observed, with peak metabolite concentrations attained 3.5 hours’ post-ingestion [56]. This finding underscores the extensive metabolism that anthocyanins undergo following oral administration.

#### 6.1.2. Animal Studies

Animal models have provided complementary evidence regarding anthocyanin bioavailability and metabolism. Matsumoto et al. [57] demonstrated that orally administered delphinidin-3-rutinoside and cyanidin-3-rutinoside are directly absorbed in rats and humans, appearing in blood as intact glycosylated forms. Following oral administration of a purified delphinidin-3-rutinoside solution (800 μmol/kg body weight) to rats, the anthocyanin was detected in the plasma with C_max_ values of 580 ± 410 nmol/L, occurring 0.5–2.0 h after administration. In human volunteers who received a mixture of black currant anthocyanins (6.24 μmol/kg body weight), delphinidin-3-rutinoside exhibited a plasma C_max_ of 73.4 ± 35.0 nmol/L at 1.25–1.75 h post-intake, with cumulative urinary excretion of 0.11 ± 0.05% of the ingested dose over 8 h [57].

In a subsequent study, Matsumoto et al. [58] calculated pharmacokinetic bioavailability of delphinidin-3-rutinoside in rats by measuring plasma concentrations following oral or intravenous administration. The anthocyanin was primarily absorbed and excreted into urine as the unmetabolized form, with T_max_ of 26.3 min and C_max_ of 0.285 ± 0.071 μmol/L. Relative to intravenous injection, oral administration resulted in complete bioavailability of 0.49 ± 0.15 [58]. Small amounts of the methylated metabolite 4′-O-methyl-delphinidin-3-rutinoside were detected in plasma, but neither aglycones nor glucuronide/sulfate conjugates were observed [58].

Wu et al. [59] investigated the impact of aglycone structure and sugar moieties on the absorption and metabolism of anthocyanin in weanling pigs fed freeze-dried berry powders. A critical finding was that delphinidin anthocyanin were not metabolized to any measurable extent, whereas cyanidin anthocyanins underwent methylation and glucuronidation. Anthocyanins with di- or trisaccharides (e.g., rutinoside or sambubioside) were excreted in urine primarily as intact forms, with over 80% remaining unmetabolized from black currant, elderberry, or Marion blackberry sources [59]. This structural specificity is significant when considering the metabolism of maqui anthocyanins, given the predominance of delphinidin glycosides with complex sugar moieties.

#### 6.1.3. Comparative Bioavailability Across Berry Sources

The bioavailability of anthocyanins exhibits significant variation depending on berry source, anthocyanin structure, and glycosylation pattern. Kay [60] conducted a review of the literature on the absorption, metabolism, and pharmacokinetics of anthocyanins in humans, noting that the maximum plasma concentrations range from 1.4 to 592 nmol/L and occur 0.5–4 h post-consumption (doses: 68–1300 mg). The mean urinary excretion of the ingested dose is reported to be between 0.03 and 4%, with elimination half-lives ranging from 1.5 to 3 h [60]. These wide ranges are indicative of variability in anthocyanin structure, food matrix effects, and inter-individual differences in metabolism.

Fang [61] addressed the apparent paradox of low plasma anthocyanin concentrations despite evidence of efficient absorption. Following oral administration of cyanidin-3-glucoside and pelargonidin-3-glucoside to human subjects, 30% and 56%, respectively, were recovered as the degradation products protocatechuic acid and 4-hydroxybenzoic acid in plasma. Furthermore, 12.4% of 13C was recovered from urine and breath following oral ingestion of [13C]-cyanidin-3-glucoside, suggesting that the actual percentage absorbed across the gastrointestinal wall could be substantially higher than apparent from intact anthocyanin measurements, due to extensive presystemic metabolism [61].

### 6.2. Intestinal Absorption Mechanisms

The mechanisms by which anthocyanins are absorbed across the intestinal epithelium remain incompletely characterized, though several pathways have been proposed. The glycosylation pattern has been identified as a critical determinant of absorption route. Matsumoto et al. [57] demonstrated that 3-O-rutinosyl anthocyanins (bearing the disaccharide rutinoside) are directly absorbed and appear in blood as intact glycosides, suggesting absorption without prior deglycosylation. This finding contradicts earlier assumptions that anthocyanins must be hydrolyzed to aglycones before absorption [57].

Hidalgo et al. [62] and Alvarado et al. [63] investigated the mechanism by which delphinidin-rich maqui berry extract (Delphinol^®^) reduces postprandial blood glucose in individuals with impaired glucose regulation. Their research identified inhibition of sodium-dependent glucose transport (SGLT) by delphinidins in the rodent jejunum. In a double-blind, placebo-controlled, crossover study of ten volunteers with moderate glucose intolerance, Delphinol^®^ intake prior to rice consumption significantly lowered postprandial blood glucose and insulin levels in comparison with the placebo [62]. In a diabetic rat model, the administration of Delphinol^®^ to rats daily over a period of four months resulted in a significant decrease in fasting blood glucose levels, which were indistinguishable from those observed in healthy non-diabetic rats [62].

Alvarado et al. [63] proceeded to investigate this mechanism in prediabetic humans by means of OGTTs. Delphinol^®^ was found to have a significant and dose-dependent effect on lowering both basal glycemia and insulinemia. The findings of the study demonstrated that lower doses were associated with a delay in the occurrence of postprandial glycemic and insulinemic peaks, while higher doses were found to reverse this tendency. The authors of the study posited three potential mechanisms: inhibition of intestinal SGLT, an incretin-mediated effect, or improved insulin sensitivity [63]. The interaction between anthocyanins and intestinal transporters may have implications not only for glucose absorption but also for anthocyanin uptake itself.

The bioaccessibility of anthocyanins, defined as the proportion of the total anthocyanins released from the food matrix that is available for absorption, is influenced by gastrointestinal conditions. Lila et al. [64] compared two complementary approaches for gauging bioavailability: an in vitro model (TIM-1) mimicking the human gastrointestinal tract from swallowing through the ileum, and in vivo rodent models with biolabeled anthocyanins. The TIM-1 model demonstrated that most anthocyanins from maqui berry and wild blueberry were bioaccessible between the second- and third-hours post-ingestion [64]. This temporal pattern corresponds with the observed plasma concentration peaks in human studies.

### 6.3. Metabolism and Plasma Metabolites

#### 6.3.1. Phase II Metabolism

Following absorption, anthocyanins undergo extensive Phase II metabolism, involving conjugation reactions such as glucuronidation, methylation, and sulfation. Agulló et al. [56] found that sulfated compounds represented approximately 83.6% of total urinary excretion following maqui-citrus beverage consumption. The predominant metabolite, TIFA-sulfate, attained a concentration of 91.16 μg/mg creatinine with sucralose as the sweetener [56]. This extensive sulfation is consistent with broader patterns of flavonoid metabolism and may influence the biological activity and tissue distribution of anthocyanin metabolites.

Kay [60] noted that while glucuronidation, methylation, and sulfation are the most cited conjugation reactions for flavonoid metabolism, evidence for extensive anthocyanin metabolism was limited until recently. New evidence suggests that anthocyanins are absorbed and transported in human serum and urine primarily as metabolites, with recent studies documenting 68–80% of anthocyanins as metabolized derivatives in human urine [60]. This metabolic transformation is significant to comprehend the bioactive forms that are responsible for health effects.

#### 6.3.2. Delphinidin-Specific Metabolic Pathways

Delphinidin anthocyanins manifest distinctive metabolic patterns when compared to other anthocyanin subclasses. As demonstrated by Wu et al. [59], delphinidin anthocyanins were not metabolized to any measurable extent in weanling pigs. This contrasts with cyanidin anthocyanins, which underwent extensive methylation and glucuronidation. This resistance to metabolism may contribute to the appearance of delphinidin glycosides as intact forms in plasma and urine [59].

However, Matsumoto et al. [58] detected minute quantities of 4′-O-methyl-delphinidin-3-rutinoside in rat plasma after the oral administration of delphinidin-3-rutinoside, thereby suggesting that a degree of methylation does indeed occur, albeit to a negligible extent. The methylated form was found to be excreted into bile more frequently than into urine, indicating that differential excretion pathways exist for parent compounds and metabolites [58].

As posited by Schön et al. [54], the breakdown PCA and GA were identified in human plasma following the ingestion of a maqui berry extract. These phenolic acids are thus considered ring-fission products, arising from the degradation of anthocyanins, either through chemical instability at physiological pH or through microbial metabolism in the colon. The relative contributions of chemical degradation versus enzymatic/microbial transformation remain areas of active investigation.

#### 6.3.3. Urinary and Biliary Excretion

The excretion routes and kinetics of anthocyanins and their metabolites provide insights into their systemic disposition. Matsumoto et al. [58] found that delphinidin-3-rutinoside was primarily excreted to urine as the intact form (795 ± 375 nmol over 8 h) and to bile as the methylated form (4′-O-methyl-delphinidin-3-rutinoside: 12.3 ± 2.91 nmol). This differential excretion pattern suggests that methylation may target anthocyanins for biliary elimination, potentially involving enterohepatic recirculation [58].

The low urinary recovery of intact anthocyanins (typically 0.03–4% of ingested dose) has been interpreted as evidence of poor bioavailability [60]. However, Fang [61] advanced the argument that this interpretation may be misleading, as it fails to account for extensive presystemic metabolism and the formation of degradation products. When metabolites and degradation products are included in the analysis, there is a substantial increase in total recovery, suggesting that absorption may be more efficient than would be indicated by measurements of intact anthocyanins alone [64].

Agulló et al. [51] conducted a study in which urinary metabolites were tracked over a 24 h period following the consumption of a maqui-citrus beverage. The collection of samples occurred at three distinct intervals: 0–3.5 h, 3.5–12 h, and 12–24 h. The highest concentrations of the metabolites in question were recorded at 3.5 h, with continued excretion of the substances being detectable up to 24 h later. The extended excretion window suggests either prolonged absorption from the lower gastrointestinal tract, enterohepatic recirculation, or release from tissue reservoirs [51].

### 6.4. Bioaccessibility and In Vitro Digestion Studies

In vitro digestion models provide controlled systems for studying the stability and bioaccessibility of anthocyanins under simulated gastrointestinal conditions. Fredes et al. [65,66] conducted extensive investigations of maqui anthocyanin stability and bioaccessibility using in vitro digestion models. Their research demonstrated that the bioaccessibility of anthocyanins from maqui juice microparticles was 10% higher than from unencapsulated maqui juice [65]. The chemical structure of anthocyanins exerts a significant influence on both encapsulation efficiency and stability. 3-O-glycosylated anthocyanins exhibit the lowest level of stability [65].

The half-life values of the anthocyanins during the storage period (160 days at 60 °C) were found to vary according to their structural characteristics. Specifically, delphinidin-3-sambubioside demonstrated a half-life of 198 days, delphinidin-3-glucoside exhibited a range of 173–182 days, and cyanidin-3-glucoside displayed a duration of 154–133 days [65]. When incorporated into yogurt, anthocyanins demonstrated half-life values of 75–69 days for delphinidin-3-sambubioside [65]. These stability data carry significant implications for the formulation and storage of maqui-based functional foods.

Viuda-Martos et al. [67] conducted a study to evaluate the protective effect of different dietary fibers on the polyphenolic profile of maqui berries during in vitro gastrointestinal digestion. The combination of maqui berry with diverse dietary fiber sources has been demonstrated to enhance the bioaccessibility index of phenolic and flavonoid compounds in all instances. Sodium carboxymethyl cellulose, xanthan gum, and guar gum was found to offer the most effective protective effects, through the means of stabilizing anthocyanin, phenolic acids, and flavonoids, because of interactions with dietary fiber [67]. This stabilization potentially provides sufficient levels for absorption during gastrointestinal digestion.

Lila et al. [64] utilized the TNO gastrointestinal model (TIM-1), a model which replicates the human GI tract from the act of swallowing to the ileum, to conduct a study on the stability and bioaccessibility of maqui berry and wild blueberry anthocyanins. The model demonstrated that most anthocyanins were bioaccessible between the second and third hours after intake [64]. However, the authors noted that TIM-1 does not include the colon, where significant microbial metabolism of anthocyanins occurs, representing a limitation of this approach.

Ribnicky et al. [68] investigated the effects of a high-fat meal matrix and protein complexation on blueberry anthocyanin bioaccessibility using TIM-1. The researchers’ findings highlighted the importance of food matrix composition in modulating anthocyanin release and stability during digestion, with implications for the optimization of delivery systems.

### 6.5. Enhancement Strategies and Chemical Challenges for Improving Anthocyanin Bioavailability

#### 6.5.1. Microencapsulation Technologies

It is evident that microencapsulation has emerged as a promising strategy for protecting anthocyanins from degradation and improving their bioaccessibility. Fredes et al. [65,66] conducted a systematic investigation into the microencapsulation of maqui juice by means of spray-drying and freeze-drying whilst comparing different encapsulating agents, including inulin, sodium alginate, and maltodextrin. However, it must be acknowledged that microencapsulation does not discriminate between beneficial and potentially undesirable compounds present in maqui extracts, which are chemically complex matrices. This means that alongside bioactive anthocyanins and polyphenols, other compounds with unknown or unwanted biological effects might also be preserved, potentially influencing the extract’s efficacy or safety profile.

Furthermore, while encapsulation techniques can enhance the chemical stability and protect maqui anthocyanins during digestion, their impact on true bioavailability (the proportion that reaches systemic circulation intact) remains limited. Additional formulation strategies such as lipid-based delivery systems, nanoemulsions, or co-encapsulation with absorption enhancers may be required to overcome these challenges and optimize the functional performance of maqui-based nutraceuticals. Future studies should prioritize evaluating the in vivo bioavailability and pharmacokinetics of encapsulated maqui compounds, to support their safe and effective incorporation into clinical and commercial applications [65,66].

The application of spray-drying, utilizing inulin as the encapsulating agent, has been demonstrated to yield optimal encapsulation efficiency, with a recorded maximum of 78.6% for delphinidin-3-sambubioside-5-glucoside [65]. It has been demonstrated that both spray-drying and freeze-drying result in the production of powders that exhibit comparable anthocyanin stability and bioaccessibility. However, it has been observed that the morphology and particle size of these powders can significantly impact their solubility, with values ranging from 70.4% to 59.5% [69]. It is noteworthy that the microencapsulation process enhanced the bioaccessibility of anthocyanins by 10% in comparison with unencapsulated maqui juice [65].

Romero-González et al. [36] investigated freeze-dried polysaccharide microcapsules, using maltodextrin and inulin as encapsulating agents. The level of concentration and the polysaccharide matrix of the encapsulating agent have been shown to have a significant effect on the retention of bioactive compounds in the microcapsules [36]. These findings emphasize the necessity of optimizing both the encapsulation technique and the choice of wall material to ensure maximum anthocyanin protection and delivery.

Nascimento et al. [69] conducted a comprehensive literature review exploring strategies to enhance anthocyanin bioavailability and bioaccessibility in food. The review emphasized that encapsulation techniques are particularly promising, as evidenced by numerous studies that have demonstrated favorable outcomes. However, the authors emphasized that the majority of studies have been confined to in vitro experiments, thereby underscoring the necessity for further in vivo investigations to achieve a comprehensive understanding of the impact of these strategies within physiological contexts [69].

Despite the promising results obtained with microencapsulation technologies, their evaluation from a chemical perspective remains challenging when applied to complex phytochemical mixtures such as maqui berry extracts. Unlike conventional drug delivery systems designed for a single active compound, maqui extracts contain multiple anthocyanins with distinct glycosylation patterns, molecular sizes, and three-dimensional conformations, which critically influence stability, encapsulation efficiency, and release behavior [70,71]. Importantly, even within delphinidin-based anthocyanins, the specific glycoside (e.g., sambubioside, glucoside, diglucoside) determines molecular stability and interaction with encapsulating matrices, limiting the direct extrapolation of results obtained from simplified systems [72]. Moreover, recent human-based evidence suggests that encapsulation strategies may not uniformly translate into improved bioavailability for polyphenolic mixtures, underscoring the need for compound-specific and structure-informed evaluations [73]. Therefore, bioavailability outcomes derived from encapsulation studies of maqui anthocyanins should be interpreted with caution, and future research should prioritize chemically defined approaches and rigorous in vivo validation.

#### 6.5.2. Food Matrix Effects

The food matrix in which anthocyanins are consumed exerts a significant influence on their stability, release, and absorption. Nascimento et al. [69] conducted a review of the extant literature on the bioaccessibility of anthocyanins in the context of co-ingestion with matrices rich in lipids and carotenoids. The review concluded that co-ingestion with such matrices improved the bioaccessibility of certain anthocyanins, while the presence of pectin and casein affected them in a variable manner. The process of cooking has been demonstrated to result in the softening of the food matrix, thereby increasing the bioaccessibility of the foodstuff [69].

Fredes et al. [65] conducted a study to evaluate the stability of maqui anthocyanin when incorporated into yogurt, determining half-life values of 75–69 days for delphinidin-3-sambubioside. The dairy matrix provided some protection, though stability was lower than in the dry encapsulated form. This application demonstrates the feasibility of incorporating maqui anthocyanins into functional dairy products while maintaining reasonable stability.

Ribnicky et al. [68] conducted a study to examine the impact of a high-fat meal matrix on the bioaccessibility of anthocyanins in blueberries. This investigation utilized the TIM-1 model as a scientific framework. Their findings indicated that meal composition, particularly fat content, can modulate anthocyanin release and stability during digestion, with implications for the optimization of consumption recommendations.

#### 6.5.3. Dietary Fiber Co-Administration

The co-administration of dietary fiber has been identified as a pragmatic approach to augmenting the stability and bioaccessibility of anthocyanins during the gastrointestinal transit. Viuda-Martos et al. [67] demonstrated that the mixture of maqui berry with various dietary fibers resulted in an enhancement of the bioaccessibility index of phenolic and flavonoid compounds in all instances following in vitro gastrointestinal digestion.

The fibers in question exhibited differential protective effects. Sodium carboxymethyl cellulose, xanthan gum, and guar gum was found to offer the most effective protection, through their interaction with dietary fiber, thereby stabilizing anthocyanin’s, phenolic acids and flavonoids [67]. It is hypothesized that the function of these gums is to increase viscosity, slow gastric emptying, and provide physical protection against degradative conditions in the stomach and small intestine.

The mechanism of fiber protection may be multifaceted, involving a variety of factors. The process of anthocyanin encapsulation or adsorption onto fiber surfaces is the first stage of the overall procedure. This is followed by modulation of the pH in the local microenvironment, reduction in oxidative degradation through antioxidant interactions, and delayed release that extends the absorption window. Further research is required to elucidate the relative contributions of these mechanisms and to optimize fiber type and concentration for maximum benefit.

#### 6.5.4. Protein Binding Approaches

Protein binding has recently been identified as a promising strategy for enhancing anthocyanin bioaccessibility and bioavailability. As posited by Wu et al. [74], a comprehensive review of protein-binding approaches was provided, with the observation that a significant proportion of animal and human clinical studies have revealed that, following the ingestion of anthocyanin-rich foods or extracts, only trace amounts (<1% of the ingested content) of anthocyanins or their predicted metabolites were detected in plasma, indicative of low bioavailability. It has been documented that the binding of protein to anthocyanins can enhance the ultimate bioactivity, bioaccessibility, and bioavailability of the anthocyanins when compared to those delivered without a protein carrier [74].

The mechanisms by which protein binding enhances bioavailability may include: (1) protection from degradation during gastric transit, (2) improved solubility and dispersion in the intestinal lumen, (3) facilitated transport across the intestinal epithelium, and (4) reduced presystemic metabolism. However, the specific proteins, binding stoichiometries, and formulation parameters that optimize these benefits remain areas of active investigation.

#### 6.5.5. Sweetener Selection

An unexpected finding from recent research is that sweetener selection in anthocyanin-containing beverages can significantly influence bioavailability. Agulló et al. [56] investigated the effect of different sweeteners (sucrose, stevia, sucralose) on anthocyanin bioavailability from maqui-citrus beverages in a double-blind, randomized, crossover study.

Sucralose was found to provide higher bioavailability for many compounds when compared to stevia and sucrose. For cyanidin anthocyanins, sucralose demonstrated 6.7% and 22.4% higher bioavailability in comparison to stevia and sucrose, respectively [56]. The authors noted that a recent study suggested a positive interaction between sucrose and phenolic compounds, inhibiting insoluble protein-proanthocyanidin aggregates and increasing bioaccessibility/bioavailability, while stevia also improved solubility and bioavailability of phenolic compounds [56].

These findings suggest that formulation decisions regarding sweeteners should consider not only taste and caloric content but also potential interactions with bioactive compounds. The mechanisms underlying sweetener effects on anthocyanin bioavailability warrant further investigation, as they may involve alterations in gastric emptying, intestinal transit time, or direct molecular interactions affecting stability and absorption.

These approaches should be regarded as emerging and complementary strategies that require systematic chemical characterization and in vivo validation, particularly in the context of complex anthocyanin mixtures such as those present in maqui berry.

### 6.6. Enhancement Strategies for Improved Bioavailability

Despite significant advances in understanding maqui anthocyanin bioavailability and metabolism, substantial knowledge gaps and methodological limitations remain:

- Limited In Vivo Validation: Many enhancement strategies, particularly microencapsulation and dietary fiber co-administration, have been evaluated primarily in in vitro digestion models [36,65,66,67,68,69]. While these models provide valuable mechanistic insights and allow for controlled comparisons, they do not fully replicate the complexity of human digestion, absorption, and metabolism. The absence of intestinal epithelial transport, hepatic first-pass metabolism, and colonic microbial transformation in most in vitro systems limits their predictive value for actual bioavailability.
- Incomplete Understanding of Absorption Mechanisms: The precise mechanisms by which anthocyanins, particularly glycosylated forms, are absorbed across the intestinal epithelium remain incompletely characterized [54,56,66]. While evidence suggests that some anthocyanins can be absorbed as intact glycosides [57], the relative contributions of passive diffusion, carrier-mediated transport, and paracellular pathways are not well defined. The potential role of SGLT in anthocyanin uptake, suggested by the glucose-lowering effects of delphinidins [62,63], requires further investigation.
- Lack of Tissue Distribution Data: Most bioavailability studies measure anthocyanin concentrations in plasma and urine, but tissue distribution and accumulation are rarely assessed [58]. Given that some biological effects may result from local concentrations in specific tissues rather than systemic exposure, understanding tissue distribution is critical for relating bioavailability to bioactivity.
- Inter-Individual Variability: Substantial inter-individual variation in anthocyanin metabolism and bioavailability has been observed [51,60], but the genetic, physiological, and microbiome factors responsible for this variation are poorly understood. Agulló et al. [51] noted inter-individual variations in metabolic traits, but systematic investigation of the determinants of this variability is lacking.
- Colonic Metabolism: Most in vitro models and many in vivo studies do not adequately address colonic microbial metabolism of anthocyanins [53]. Given that a substantial fraction of ingested anthocyanins reaches the colon, where they undergo extensive microbial transformation to phenolic acids and other metabolites, this represents a significant gap in understanding their fate and biological activity.
- Bioavailability-Bioactivity Relationship: The apparent paradox between low systemic bioavailability (<1%) and documented health effects remains unresolved [60,75]. Whether biological effects result from low concentrations of intact anthocyanins, higher concentrations of metabolites, local effects in the gastrointestinal tract, or indirect mechanisms (e.g., modulation of gut microbiota) requires clarification.
- Limited Delphinidin-Specific Data: While maqui berry is rich in delphinidins, much of the mechanistic understanding of anthocyanin bioavailability derives from studies of cyanidin and other anthocyanin subclasses. The distinctive metabolic profile of delphinidins, particularly their resistance to extensive metabolism [59] suggests that findings from other anthocyanins may not fully apply to maqui berry.
- Standardization and Comparability: Variability in study designs, anthocyanin doses, food matrices, analytical methods, and reporting metrics makes cross-study comparisons challenging. The development of standardized protocols for bioavailability assessment would facilitate meta-analyses and more robust conclusions.

**Figure 2:**
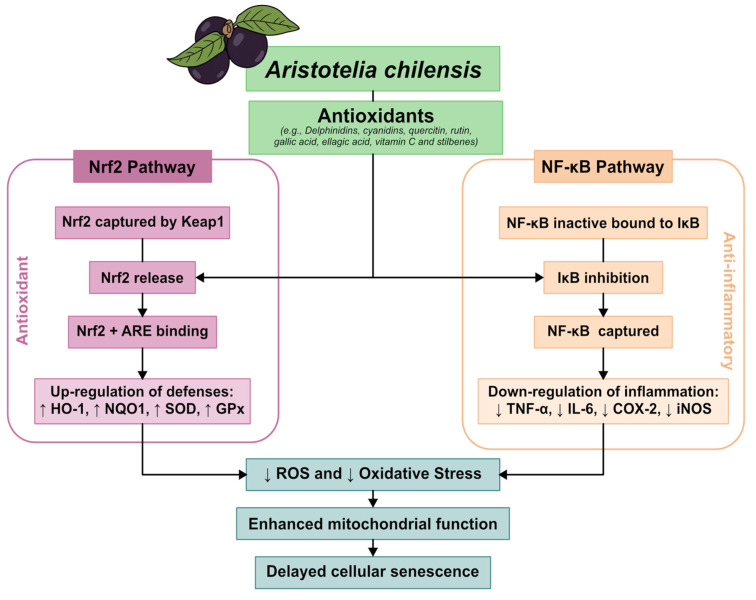
Integrative model of the molecular mechanisms underlying the bioactivity of *Aristotelia chilensis* (Maqui). This schematic summarizes the main molecular pathways associated with the antioxidant and anti-inflammatory effects of maqui. Although *Aristotelia chilensis* contains a complex mixture of polyphenolic compounds, the mechanisms depicted are primarily supported by experimental evidence for delphinidin-based anthocyanins, which represent the predominant and most bioactive fraction of the fruit. These compounds modulate redox homeostasis through activation of the Nrf2–ARE pathway, enhancement of endogenous antioxidant defenses, inhibition of NF-κB signaling, reduction in reactive oxygen species, and attenuation of pro-inflammatory cytokine expression.

For further explanation, Figure 2 below shows an integrative model of the molecular mechanisms underlying the bioactivity of *Aristotelia chilensis*.

## 7. Health Applications and Clinical Evidence

The growing burden of chronic non-communicable diseases has intensified interest in dietary strategies capable of modulating oxidative stress, inflammation, and metabolic dysfunction. In this context, functional foods rich in polyphenols have gained attention due to their capacity to interact with key molecular pathways involved in disease pathophysiology. Maqui owing to its exceptional anthocyanin content and antioxidant potential, has been increasingly investigated for its health-related applications across cardiometabolic, endocrine, and renal conditions. The following subsections summarize and critically examine the available experimental and clinical evidence supporting the health effects of maqui, with emphasis on mechanistic plausibility and translational relevance.

### 7.1. Cardiometabolic Health

Some non-communicable diseases, including CVD, may be more effectively prevented through lifestyle modifications, particularly the adoption of healthy dietary patterns. In this context, numerous studies have highlighted the importance of natural antioxidants present in fruits and vegetables as key contributors to cardiovascular protection [76].

Platelets play a critical role in the development of atherosclerosis and in the pathophysiology of thrombotic events. Evidence indicates that certain bioactive compounds found in fruits and vegetables, when consumed regularly, can inhibit platelet aggregation and thereby contribute to a reduced risk of CVD [77].

Despite its rich composition and abundance of bioactive compounds with potential antiplatelet activity, the scientific evidence directly linking maqui to antiplatelet effects remains limited.

Nevertheless, although research on the antiplatelet activity of maqui is relatively recent, its phytochemical profile strongly suggests that the presence of compounds such as anthocyanins, flavonoids, and phenolic acids may confer significant antiplatelet properties with potential cardiovascular relevance [76].

Accordingly, chemical characterization studies of maqui have identified several compounds with known antiplatelet activity, including caffeic acid, quercetin, isorhamnetin, kaempferol, and rutin, among others. Variations in the composition and concentration of phenolic compounds have been observed among different varieties, supporting the notion that extracts derived from distinct genotypes and different parts of the plant, such as immature versus mature fruits, exhibit significant differences in antiplatelet activity. Furthermore, Pearson correlation analyses have been employed to assess the contribution of individual bioactive compounds, demonstrating that phenolic compound levels are largely responsible for the antiplatelet effects attributed to maqui [78].

In addition, several studies have investigated the antioxidant activity of methanolic extracts of maqui fruit and their cardioprotective effects in in vivo models of acute ischemia/reperfusion injury in rats. In these models, methanolic maqui extracts provided significant protection against cardiac damage by reducing the incidence of reperfusion-induced arrhythmias and promoting the recovery of sinus rhythm [76].

### 7.2. Effect on Diabetes Mellitus

Type 2 diabetes represents a major public health challenge, with its global prevalence increasing steadily over the past four decades. This rise has been largely attributed to changes in environmental conditions and lifestyle patterns, characterized by declining dietary quality and a marked increase in sedentary behaviors [79].

Accumulating evidence indicates that diets rich in polyphenols, particularly those containing anthocyanins, may reduce the risk of developing type 2 diabetes. In clinical trials involving individuals with diabetes, anthocyanin supplementation has been associated with significant reductions in blood glucose concentrations and HbA1c, along with improvements in insulin secretion and insulin sensitivity [80].

Several studies have identified beneficial effects of dietary polyphenolic compounds, including anthocyanins, on glucose homeostasis. These effects are mediated through multiple potential mechanisms involving key metabolic organs such as the intestine, liver, skeletal muscle, adipose tissue, and pancreatic β-cells, as well as through interactions with the gut microbiota [81].

In this context, anthocyanins have been shown to attenuate hyperglycemia and insulin resistance through mechanisms that include inhibition of carbohydrate-digesting enzymes such as α-amylase and α-glucosidase, enhancement of glucose-stimulated insulin secretion by pancreatic β-cells, and modulation of hepatic function to improve insulin sensitivity. Additionally, anthocyanins may contribute to the reduction in hyperuricemia, a metabolic condition closely associated with hyperglycemia and insulin resistance [82].

Notably, the metabolic effects of anthocyanins appear to be further enhanced in the presence of pharmacological therapy. In murine models, combined administration of anthocyanins (100 mg/kg/day) and metformin (50 mg/kg/day) resulted in significant improvements in blood glucose levels, insulin resistance, and organ damage while also increasing the abundance of beneficial gut bacteria and the production of short-chain fatty acids [83].

Delphinol^®^, a nutritional supplement that has demonstrated the ability to naturally modulate postprandial glycemia, primarily through inhibition of the sodium–glucose cotransporter in the small intestine [84].

Consistent with these findings, a study conducted in ten volunteers with moderate glucose intolerance, using a randomized, double-blind, placebo-controlled crossover design, demonstrated the effect of Delphinol^®^, on postprandial glucose responses. Supplementation significantly inhibited undesirable increases in postprandial blood glucose levels at 60 and 90 min following the ingestion of boiled rice [85].

### 7.3. Effects on Chronic Kidney Disease

CKD constitutes a highly prevalent public health problem with a growing global impact, associated with increased cardiovascular morbidity and mortality and a progressive deterioration in quality of life. From a pathophysiological perspective, CKD is characterized by persistent oxidative stress, chronic low-grade inflammation, endothelial dysfunction, and metabolic disturbances, all of which decisively contribute to the progression of renal damage and the heightened cardiovascular risk observed in this population [86,87,88,89,90]. In contrast to cardiometabolic health and diabetes, where clinical studies have reported beneficial effects of maqui berry extracts, evidence supporting their use in CKD remains limited and largely indirect.

Oxidative stress represents a central axis in CKD progression and is evident from the early stages of the disease. The overproduction of ROS, together with impairment of endogenous antioxidant defense systems, promotes oxidative damage to lipids, proteins, and DNA, accelerating the loss of functional nephron mass and inducing glomerular and tubulointerstitial structural alterations [86,87,88]. This redox imbalance is further intensified in advanced stages and during renal replacement therapies, including hemodialysis, peritoneal dialysis, and kidney transplantation, where uremia-related factors, bioincompatibility of dialytic materials, recurrent ischemia–reperfusion events, and metabolic disturbances exacerbate ROS generation [87,90].

In the specific context of hemodialysis, patients exhibit a pronounced state of oxidative stress and persistent systemic inflammation, arising both from the uremic milieu and from intrinsic factors related to the dialysis procedure itself, such as immune activation induced by blood–membrane contact, volume fluctuations, and loss of antioxidant micronutrients. This pro-oxidative environment is associated with endothelial dysfunction, lipid peroxidation, and an elevated cardiovascular risk, which represents the leading cause of mortality in this population [87,90]. In this setting, nutritional modulation of oxidative stress through antioxidant bioactive compounds becomes particularly relevant, given its potential to attenuate pro-inflammatory signaling and partially restore impaired redox homeostasis [90].

Concurrently, sustained chronic inflammation plays a determinant role in CKD pathophysiology. Persistent activation of pro-inflammatory pathways, such as NF-κB and the NLRP3 inflammasome, together with increased circulating levels of cytokines including TNF-α, IL-6, and IL-1β, contributes to endothelial dysfunction, progressive cellular injury, and activation of profibrotic processes culminating in renal fibrosis [86,88,89]. Fibrosis represents the final common pathway of CKD and is closely associated with the activation of mediators such as TGF-β, α-SMA, and type IV collagen, which have been extensively described in experimental models of diabetic nephropathy and other CKD etiologies [86,88].

These mechanisms are particularly relevant in kidney transplantation, where oxidative stress and inflammation are central contributors to both acute ischemia–reperfusion injury and chronic allograft dysfunction. Excessive ROS generation, mitochondrial dysfunction, and activation of inflammatory cascades during the perioperative period promote tubular injury, apoptosis, and initiation of profibrotic pathways. In the long term, sustained exposure to immunosuppressive agents may further perpetuate redox imbalance and contribute to progressive deterioration of graft function [87,88,89].

From a nutritional perspective, CKD is also associated with disturbances in energy and protein metabolism, systemic inflammation, and alterations in the gut microbiota, all of which amplify oxidative stress and accelerate renal disease progression. Current evidence highlights individualized medical nutrition therapy as a fundamental pillar in the comprehensive management of CKD, not only to slow disease progression but also to modulate key metabolic, inflammatory, and oxidative processes [90]. In this context, dietary patterns rich in antioxidant bioactive compounds, particularly those of plant origin, have demonstrated favorable effects on cardiometabolic profile, systemic inflammation, and redox homeostasis [90].

Anthocyanins, a class of polyphenols widely distributed in fruits and vegetables, have emerged as compounds of particular interest due to their antioxidant, anti-inflammatory, and metabolism-modulating properties. Preclinical studies have shown that specific anthocyanins, such as cyanidin-3-O-glucoside, improve renal function, reduce glomerular fibrosis, and attenuate structural renal damage through activation of the Nrf2/HO-1 pathway and regulation of key metabolic routes, including amino acid metabolism [86]. These mechanisms are especially relevant in CKD, where Nrf2 activation is associated with enhanced expression of endogenous antioxidant enzymes and reduced renal oxidative damage [86,89].

Maqui stands out for its exceptionally high anthocyanin content, particularly delphinidin-based anthocyanins, which confer remarkable antioxidant capacity. Although most available evidence derives from experimental studies and clinical investigations in cardiometabolic conditions, the underlying mechanisms—reduction in oxidative stress, inhibition of inflammatory pathways, and modulation of cellular metabolism—are highly relevant to CKD pathophysiology, including patients undergoing hemodialysis and kidney transplantation [86,87,88,89]. Moreover, contemporary CKD management increasingly emphasizes plant-based dietary patterns rich in antioxidant bioactive compounds as a strategy to modulate inflammation, oxidative stress, and CKD-associated cardiovascular risk [90].

Nevertheless, despite strong biological plausibility and supportive preclinical evidence, direct clinical data evaluating the efficacy and safety of maqui berry extracts or specific anthocyanins in CKD patients are currently lacking. To date, no randomized, adequately powered clinical trials have specifically assessed maqui supplementation across different CKD stages or in populations undergoing renal replacement therapies. Therefore, existing evidence should be considered hypothesis-generating rather than confirmatory. Well-designed controlled clinical studies are required to evaluate efficacy, safety, bioavailability, and long-term outcomes of anthocyanin-rich interventions in CKD [87,88,89,90].

Overall, the integration of nutritional strategies based on antioxidant-rich functional foods, such as maqui, represents a promising avenue for translational research in nephrology. Any potential application should be regarded as complementary to conventional medical nutrition therapy and implemented within an individualized framework supported by robust clinical evidence [90].

## 8. Food Technology, Functional Foods and Product Development

The field of food technology has evolved significantly over recent decades, driven by consumer demand for foods that not only provide basic nutrition but also confer health benefits beyond essential nutrients. This paradigm shift has given rise to the development of functional foods, which are foods formulated to deliver specific physiological benefits that may reduce the risk of chronic disease or promote optimal health [91]. Within this context, the integration of exotic fruits and berries rich in bioactive compounds, such as maqui, represents a promising area of product innovation.

Functional foods are characterized by the presence of bioactive components, such as polyphenols, flavonoids, anthocyanins, and dietary fibers, which exhibit biological activities, including antioxidant, anti-inflammatory, and cardiometabolic regulatory effects [92,93]. Maqui has been the subject of extensive phytochemical research, demonstrating one of the highest total phenolic contents among berries, especially in terms of anthocyanin concentration [94,95,96]. These properties have stimulated interest in incorporating maqui into food formulations, nutraceuticals, and dietary supplements aimed at preventive health applications.

### 8.1. Technological Considerations for Maqui Incorporation

Incorporating maqui into food products poses several technological challenges and opportunities. Maqui extracts, juices, powders, and encapsulated forms must maintain stability of bioactives during processing, storage, and digestion [97]. Studies have shown that high temperatures, prolonged heating, and exposure to light can degrade anthocyanins and reduce antioxidant capacity, necessitating optimized processing conditions such as mild thermal treatments, microencapsulation technologies, and the use of stabilizing carriers [98,99].

Microencapsulation, for example, has emerged as a robust strategy to enhance the stability and bioavailability of maqui bioactives. Techniques such as spray drying, freeze-drying and complex coacervation can protect sensitive compounds like anthocyanins from oxidation and degradation, resulting in functional ingredients that can be incorporated into beverages, yogurts, and bakery products without significant loss of activity [100,101].

### 8.2. Development of Functional Products

The development of maqui-enriched functional products has progressed across various food matrices. Beverages fortified with maqui extracts have shown increased antioxidant activity and consumer appeal due to their vibrant color and perceived health benefits [102]. Dairy products, such as probiotic yogurts enriched with maqui powder or extract, have been reported to exhibit enhanced phenolic content and antioxidant capacity, with potential synergistic effects between probiotic metabolic activity and polyphenol bioactivity [103].

Moreover, solid food applications, including cereal bars and snack foods, have leveraged maqui’s sensory characteristics and functional profile to create products targeting specific health niches, such as metabolic health and oxidative stress reduction. Sensory optimization and consumer acceptance studies suggest that balancing maqui concentration with flavor masking and texture enhancement is critical for market success [104,105].

### 8.3. Regulatory and Safety Aspects

Product development must also consider regulatory frameworks that govern health claims, ingredient safety, and labeling. In many jurisdictions, functional foods containing bioactive ingredients must undergo safety evaluation and substantiation of health claims through clinical evidence [91,106]. For maqui, emerging clinical studies support claims related to antioxidant effects and cardiometabolic outcomes, but further high-quality randomized controlled trials are needed to substantiate specific health claims for regulatory approval [107].

## 9. Safety, Toxicology and Dosage Considerations

The safety profile, toxicological evidence, and dosage recommendations for maqui are key considerations when evaluating its potential functional and therapeutic use. Although maqui has a long history of food use in Chile and Argentina, systematic toxicological evaluations remain limited, and existing data derive mainly from preliminary clinical and in vitro studies rather than formal toxicity trials.

### 9.1. Safety and Toxicological Evidence

Overall, clinical reports and available safety assessments indicate a favorable safety profile for maqui berry extracts at typical dietary or supplemental doses. A systematic review of botanical dietary supplements noted that, to date, no dedicated preclinical toxicity studies on maqui consumption have been reported in the scientific literature, underscoring a significant gap in formal toxicological evaluation [108]. This finding suggests that while preliminary evidence does not indicate overt toxicity, rigorous safety studies are still required.

Clinical intervention trials with standardized maqui extracts, such as Delphinol^®^, have reported no significant adverse effects at studied doses in adult participants. For example, in a randomized, placebo-controlled trial assessing oxidative stress biomarkers, 450 mg/day of a standardized maqui extract (equivalent to ~162 mg anthocyanins) administered for 4 weeks was well tolerated, with no significant adverse events or safety concerns reported in healthy and overweight adults [109].

Exploratory studies using higher intake levels (e.g., 2 g of maqui extract twice daily for two weeks) showed no reported digestive symptoms, adverse events, or safety issues in adult smokers, indicating tolerance of elevated polyphenol intake in the short term [110]. These studies suggest that maqui extracts can be tolerated at doses higher than those typically found in food sources without immediate negative effects.

In vitro studies also support a baseline safety perspective, showing that maqui extracts did not produce cytotoxic effects on epithelial and macrophage cell lines at functional concentrations and that cellular viability was maintained, supporting the absence of major toxicity in vitro [111].

### 9.2. Dosage Considerations

Clear dosage guidelines for maqui are still embryonic, given the limited number of controlled human studies. Existing clinical interventions often use standardized anthocyanin-rich extracts, with the most studied dose being 450 mg/day of Delphinol^®^ for 4 weeks, which has been associated with improvements in oxidative stress markers and was well tolerated [109].

Commercial safety databases note that oral maqui supplementation up to approximately 180 mg/day for up to 3 months is considered possibly safe, although these recommendations are not based on extensive clinical evidence and should be interpreted with caution due to limited long-term safety data [52]. Higher doses (e.g., grams per day) have been used in small exploratory studies but without comprehensive safety monitoring protocols.

Specific populations, such as pregnant or lactating women, lack sufficient safety evidence; existing summaries explicitly state that reliable information is not available and thus use should be approached with caution or avoided until further research clarifies safety in these groups [52,112].

### 9.3. Drug Interactions and Special Populations

Interactions between maqui berry extracts and medications remain under-investigated. Some reports suggest that maqui’s potential to affect glycemic responses could theoretically interact with antidiabetic medications due to its influence on blood glucose dynamics, though this has not been conclusively studied in formal interaction trials [52,112].

Allergic reactions to berries are rare but can occur with any botanical; specific case reports of hypo- or hypersensitivity reactions to maqui are anecdotal and not documented extensively in clinical literature.

## 10. Conclusions

Maqui emerges as a Chilean functional food of high potential relevance due to its exceptional antioxidant and anti-inflammatory properties, primarily driven by its high content of delphinidin-based anthocyanins and other polyphenolic compounds. Accumulated evidence indicates that maqui bioactives modulate key molecular pathways involved in redox homeostasis and inflammatory regulation, including activation of the Nrf2–ARE axis, upregulation of endogenous antioxidant enzymes, inhibition of NF-κB signaling, reduction in reactive oxygen species, and preservation of mitochondrial function.

Preclinical studies consistently support the cardiometabolic, anti-inflammatory, and cytoprotective effects of maqui extracts, with emerging evidence suggesting biologically plausible nephroprotective actions through attenuation of oxidative stress, inflammation, and fibrotic processes relevant to chronic kidney disease. Early human studies further report beneficial effects on glycemic control, endothelial function, and systemic oxidative stress, reinforcing the translational interest of maqui-derived bioactives. However, it should be noted that most available clinical data remain preliminary and are largely derived from small-scale or short-term interventions.

Despite strong biological plausibility and robust mechanistic evidence, several challenges remain. The low apparent systemic bioavailability of anthocyanins, extensive presystemic metabolism, and variability related to the food matrix, formulation, and inter-individual responses underscore the need for optimized and chemically informed delivery strategies. Advances in microencapsulation, food matrix design, and co-administration with dietary fibers or proteins represent promising, yet still emerging, approaches to enhancing bioaccessibility and biological efficacy.

In conclusion, maqui represents a valuable model of a food-derived antioxidant intervention with potential relevance for cardiometabolic, vascular, and renal health. Its incorporation into functional foods or nutraceutical formulations should be regarded as a complementary strategy within evidence-based nutritional interventions rather than a standalone therapeutic approach. Nevertheless, well-designed, adequately powered clinical trials are required to confirm efficacy, establish dose–response relationships, assess long-term safety, and define its therapeutic potential in chronic non-communicable diseases. Addressing these gaps will be essential to translate the rich phytochemical profile of maqui into clinically meaningful health applications.

## Figures and Tables

**Table 1:** Polyphenolic Composition of *Aristotelia chilensis* (Maqui) Fruit.

Compound Class	Specific Compound	Reported Content	Unit	Fruit Matrix/Condition
Total phenolics	Total phenolics (gallic acid equivalents)	12.3–15.8	g GAE·kg^−1^ FW	Ripe fruit, wild ecotypes
Total anthocyanins	Total anthocyanins (cyanidin-3-glucoside equivalents)	6.2–12.0	g C3G·kg^−1^ FW	Ripe fruit
Individual anthocyanins	Delphinidin-3-sambubioside-5-glucoside	28–35	mg·g^−1^ DW	Freeze-dried fruit
	Delphinidin-3,5-diglucoside	18–25	mg·g^−1^ DW	Freeze-dried fruit
	Cyanidin-3-sambubioside-5-glucoside	6–10	mg·g^−1^ DW	Freeze-dried fruit
Flavonols	Quercetin derivatives	0.4–0.9	mg·g^−1^ DW	Fruit
	Myricetin derivatives	0.3–0.7	mg·g^−1^ DW	Fruit
Phenolic acids	Gallic acid	0.2–0.4	mg·g^−1^ DW	Fruit
	Protocatechuic acid	0.15–0.35	mg·g^−1^ DW	Fruit
Antioxidant capacity	FRAP	130–170	mmol Fe^2+^·kg^−1^ FW	Fresh fruit
	DPPH (EC_50_)	1.3–1.7	mg FW	Fresh fruit

**Table 2:** Comparative biological properties of maqui (*Aristotelia chilensis*) and other anthocyanin-rich berries.

Species	Biological Properties
Maqui	Several health benefits and bioactivities of maqui berry extracts have been reported, including inhibition of low-density lipoprotein (LDL) oxidation, skin photoprotective and anti-photoaging effects, antimicrobial and anticancer activities, antihemolytic protection, prevention of atherosclerosis, cardioprotective effects, obesity control, inhibition of adipogenesis, and improvement of diabetes-related symptoms [3].
Acaí	Extracts from açaí fruit have been shown to exert antioxidant and anti-inflammatory actions associated with the prevention and treatment of risk factors for diabetes, dyslipidemia, hypertension, and cardiovascular diseases. In addition, açaí has demonstrated anticancer, antiatherogenic, antimicrobial, antinociceptive, anticonvulsant, antileishmanial, and anti-aging activities [23].
Calafate	The importance of Calafate anthocyanins lies in their protective effects against chronic diseases and their potential role in the prevention of degenerative disorders. Similarly to maqui, Calafate has shown an improvement in glucose uptake, suggesting beneficial effects on glucose metabolism [24].
Cranberry/Blueberry	Cranberries are among the few fruits with a high content of proanthocyanidins, which inhibit the adhesion of *Escherichia coli* to the urinary tract. Consumption of cranberries has been associated with the prevention of dental caries and periodontal diseases, inhibition of urinary tract infections, reduction in inflammation, maintenance of digestive health, and lowering of cholesterol levels [25,26].

## Data Availability

No new data were created or analyzed in this study. Data sharing is not applicable to this article.

## Abbreviations

The following abbreviations are used in this manuscript:

ROS	Reactive oxygen species
Nrf2-ARE	Nuclear factor erythroid 2–related factor 2–Antioxidant Response Element
Nrf2	Nuclear factor erythroid 2–related factor 2
ARE	Antioxidant response elements
NF-κB	Nuclear factor kappa B
NO	Nitric oxide
eNOS	Endothelial nitric oxide synthase
DPPH	2,2-Diphenyl-1-picrylhydrazyl
ORAC	Oxygen Radical Absorbance Capacity
ABTS	2,2′-Azino-bis (3-ethylbenzothiazoline-6-sulfonic acid)
AMPK	AMP-activated protein kinase
CVD	Cardiovascular disease
TNF-α	Tumor Necrosis Factor-α
IL-6	Interleukin-6
TGF-β	Transforming growth factor-β
IL-1β	Interleukin-1β
α-SMA	α-smooth muscle actin
COX-2	Cyclooxygenase-2
HO-1	Heme Oxygenase-1
NQO1	NAD(P)H quinone oxidoreductase 1
SOD	Superoxide Dismutase
GPx	Glutathione Peroxidase
Keap1	Kelch-like ECH-associated protein 1
CSE	Cigarette smoke extract
MBE	Maqui berry extract
TNBS	Trinitrobenzene sulfonic acid
DCF	Dichlorofluorescein
MDA	Malondialdehyde
H_2_O_2_	Hydrogen peroxide
UVB	Ultraviolet B
IKK	IκB kinase
PGE_2_	Prostaglandin E_2_
iNOS	Inducible Nitric Oxide Synthase
ASC	Apoptosis-associated speck-like protein containing a CARD
PGC-1α	Peroxisome proliferator-activated receptor gamma coactivator-1 alpha
TFAM	Transcription Factor A, Mitochondrial
SASP	Senescence-associated secretory phenotype
DNA	Deoxyribonucleic acid
SA-β-gal	β-galactosidase
LC-MS/MS	Liquid chromatography-tandem mass spectrometry
UHPLC-ESI-MS/MS	Ultra-High-Performance Liquid Chromatography coupled to Electrospray Ionization Tandem Mass Spectrometry
DG	Digoxin
CS	Corticosterone
SGLT	Sodium-dependent glucose transport
OGTTs	Oral glucose tolerance tests
TIM-1	T-cell immunoglobulin and mucin-domain containing-1
TIFA-sulfate	Sulfated TIFA (trihydroxyisoflavone A) metabolite
PCA	Protocatechuic acid
GA	Gallic acid
HbA1c	Glycated hemoglobin
CKD	Chronic Kidney Disease
FW	Fresh weight
DW	Dry weight
GAE	Gallic acid equivalents
C3G	Cyanidin-3-glucoside equivalents
UAE	Ultrasound-assisted extraction
MAE	Microwave-assisted extraction
